# Adherence to a Web-Based Physical Activity Intervention for Patients With Knee and/or Hip Osteoarthritis: A Mixed Method Study

**DOI:** 10.2196/jmir.2742

**Published:** 2013-10-16

**Authors:** Daniël Bossen, Michelle Buskermolen, Cindy Veenhof, Dinny de Bakker, Joost Dekker

**Affiliations:** ^1^Netherlands Institute for Health Services Research (NIVEL)UtrechtNetherlands; ^2^Tilburg UniversityTranzoTilburgNetherlands; ^3^VU University Medical Center AmsterdamDepartment of Rehabilitation Medicine & Department of Psychiatry/ EMGO InstituteAmsterdamNetherlands

**Keywords:** adherence, usage, Web-based intervention, mixed method study

## Abstract

**Background:**

Web-based interventions show promise in promoting a healthy lifestyle, but their effectiveness is hampered by high rates of nonusage. Predictors and reasons for (non)usage are not well known. Identifying which factors are related to usage contributes to the recognition of subgroups who benefit most from Web-based interventions and to the development of new strategies to increase usage.

**Objective:**

The aim of this mixed methods study was to explore patient, intervention, and study characteristics that facilitate or impede usage of a Web-based physical activity intervention for patients with knee and/or hip osteoarthritis.

**Methods:**

This study is part of a randomized controlled trial that investigated the effects of Web-based physical activity intervention. A total of 199 participants between 50-75 years of age with knee and/or hip osteoarthritis were randomly assigned to a Web-based intervention (n=100) or a waiting list (n=99). This mixed methods study used only data from the individuals allocated to the intervention group. Patients were defined as users if they completed at least 6 out of 9 modules. Logistic regression analyses with a stepwise backward selection procedure were executed to build a multivariate prediction usage model. For the qualitative part, semistructured interviews were conducted. Both inductive and deductive analyses were used to identify patterns in reported reasons for nonusage.

**Results:**

Of the 100 participants who received a password and username, 46 completed 6 modules or more. Multivariate regression analyses revealed that higher age (OR 0.94, *P*=.08) and the presence of a comorbidity (OR 0.33, *P*=.02) predicted nonusage. The sensitivity analysis indicated that the model was robust to changes in the usage parameter. Results from the interviews showed that a lack of personal guidance, insufficient motivation, presence of physical problems, and low mood were reasons for nonusage. In addition, the absence of human involvement was viewed as a disadvantage and it negatively impacted program usage. Factors that influenced usage positively were trust in the program, its reliability, functionality of the intervention, social support from family or friends, and commitment to the research team.

**Conclusions:**

In this mixed methods study, we found patient, intervention, and study factors that were important in the usage and nonusage of a Web-based PA intervention for patients with knee and/or hip osteoarthritis. Although the self-guided components offer several advantages, particularly in relation to costs, reach, and access, we found that older patients and participants with a comorbid condition need a more personal approach. For these groups the integration of Web-based interventions in a health care environment seems to be promising.

**Trial Registration:**

The Netherlands National Trial Register (NTR): NTR2483; http://www.trialregister.nl/trialreg/admin/rctview.asp?TC=2483 (Archived by Webcite at http://www.webcitation.org/67NqS6Beq).

## Introduction

Osteoarthritis (OA) in the knee or hip is a prevalent musculoskeletal disorder characterized by joint pain, joint stiffness, and functional disability [1]. Regular physical activity (PA) has been recognized as an effective lifestyle strategy in the nonpharmacological management of knee and hip OA [2,3]. Despite recommendations, people with knee or hip OA are less physically active than the general population [4,5].

In an attempt to enhance a physically active lifestyle in patients with knee and/or hip OA, we developed a Web-based PA intervention. The intervention, entitled *Join2move*, is a self-paced 9-week PA program in which the patient’s favorite recreational activity is gradually increased during fixed time periods. In a recent randomized controlled trial (RCT) among 199 participants with knee and/or hip OA [6], *Join2move* was demonstrated to be effective compared to a waiting list control group. Besides enhanced levels of PA, this study showed significant improvements in physical functioning, self-efficacy, pain levels, tiredness, and anxiety in the intervention group.

Unfortunately, substantial rates of nonusage were observed. A considerable proportion of potential users was never exposed to important program content. This is consistent with other studies [7-16]. For example, two studies [15,16] testing a Web-based PA intervention reported that 60% of their diabetes patients accessed the website once a week. The issue of nonusage is described in Eysenbach’s Law of Attrition [17]. According to Eysenbach, characteristics related to the participant, intervention, and study may play a pivotal role in the adoption or rejection of Web-based interventions. Studies have demonstrated that older age groups [10,18-22], people with a healthy lifestyle [10,20], those with social ties [23], higher educated patients [22], and women [22,24] are more likely to adhere to Web-based interventions. In addition to user characteristics, the characteristics of the intervention itself can also influence usage. For instance, self-guided interventions with minimal human “push factors” (eg, online counseling or emails) show higher rates of nonusage than programs with substantial human involvement [17,25,26]. Other intervention characteristics that predict usage are program duration and complexity. Generally, shorter, more concise interventions achieve better usage rates compared with more extensive interventions [27]. Moreover, it is known that study-related factors (eg, attention, commitment, and a belief in the importance of research), especially in RCTs [26], are positively related to usage [18,28].

Although considerable research has been devoted to quantitative predictors of nonusage, little qualitative research has been conducted on the underlying reasons for nonusage. Therefore, we conducted a mixed methods study to gain a deeper understanding of actual usage patterns, possible attrition predictors, and reasons for (non)usage. This is a necessary step toward enhancing program usage and may help us to make the *Join2move* intervention even more effective.

In this study, we utilized a mixed methods design employing both quantitative and qualitative (interviews) methods. By integrating the quantitative and qualitative results, we aimed to identify patient-, intervention-, and study-related characteristics that may facilitate or impede the usage of Web-based intervention for patients with knee and/or hip OA. Since this study was explorative by nature, no a priori hypotheses were formulated.

## Methods

### Study Design and Participants

Data from this study were retrieved from a randomized controlled trial that aimed at evaluating the effectiveness of the *Join2move* intervention for patients with hip and/or knee OA [6]. In brief, the design of the study was a randomized, nonblinded, controlled, two-arm trial. Ethical approval was obtained from the medical ethics committee of the VU University Medical Center Amsterdam. Enrollment started on January 3, 2011 and ended on November 5, 2011. Sedentary volunteers with knee and/or hip OA were recruited via articles in newspapers and health-related websites. The eligibility criteria for participants were (1) aged 50-75, (2) self-reported OA in knee and/or hip, (3) self-reported inactivity (<30 minutes of moderate PA less than 5 days in a week), (4) no face-to-face consults for OA with a health care provider, other than general practitioner, in the last 6 months, (5) ability to access the Internet weekly, and (6) no contra-indications to exercise without supervision. In total, 199 eligible participants were randomly assigned either to the intervention (n=100) or waiting list control group (n=99). Baseline, 3-month, and 12-month follow-up data were collected via online questionnaires. Primary outcomes were PA, physical functioning, and self-perceived effect. Self-perceived effect was assessed by asking participants about the degree of change since their previous assessment (much worse to much better*).* Both short-term and long-term results revealed positive effects of *Join2move* with respect to PA, physical functioning, self-perceived effect, and several other secondary outcomes [6].

### Intervention

**Figure 1:**
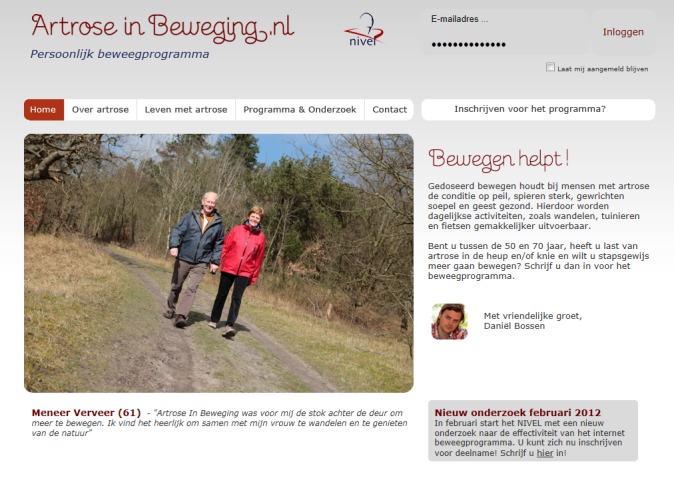
Homepage Join2move.

Over the course of 1 year, experts from the Netherlands Institute for Health Services Research developed the *Join2move* intervention. The *Join2move* intervention is based on a previously developed and evaluated behavioral graded activity (BGA) program for patients with knee and/or hip OA [29]. Details of the *Join2move* intervention and the development process are described in another publication [30]. In brief, the *Join2move* intervention is a fully automated Web-based intervention that contains automatic functions (automatic messages on the website and automatic emails) without human support. Screenshots illustrating different stages of the Join2move program are presented in Multimedia Appendix 1. Participants are presented with the homepage (see Figure 1). *Join2move* is a self-paced 9-week PA program in which the patient’s favorite recreational activity is gradually increased in a time-contingent manner (ie, on fixed time points). In the first week, users select a central activity such as cycling or walking and perform a 3-day self-test. Based on the performance from the self-test, a range of goals is automatically generated and presented on the website. In this way, achievable goals are set. Users have the option to choose one of the proposed short-term goals between a lower and upper limit. Depending on the selected goal, 8 tailored modules are generated and presented weekly on the website. Modules remain on the website for 1 week. After 7 days, users are presented with an evaluation form about pain and performance. Pain is assessed on a 10-point Numerical Rating Scale (0 is no pain, 10 is worst possible pain). Performance is measured by three items: (1) “I completed the module as instructed”, (2) “I did more than the instructed module”, and (3) “I did less than the instructed module” (due to time constraints, weather conditions, pain in my knee and/or hip, or other physical complaints). Subsequently, tailored to the answers from the evaluation form, automated text-based messages are generated. If users indicated that a module was missed due to time constraints or weather conditions, they had the option to repeat the current module or to continue with the next module. When users indicated that a module was missed due to pain in knee and hip or other physical complaints, they had the option to repeat the module (a maximum of three times), adapt the intensity of the module, or proceed to the next module. Since personal messages are updated on a weekly basis, users are encouraged to log in once a week. Automated emails are generated if participants do not log on the website for 2 weeks. At the end of the program, the website presents a motivational message to perform regular PA in the future. In total, the program lasted 9 weeks.

### Data Collection and Outcomes of the Quantitative Study

#### Overview

Program usage (ie, the number of completed program modules) was monitored throughout the intervention period. A module consisted of a text-based assignment plus accompanying evaluation form, which was presented on the website for 7 consecutive days. Once a participant had filled out the evaluation form 7 days after receiving the weekly assignment, the module was defined as completed and the user was automatically presented with a new weekly assignment. If a scheduled weekly module was missed, participants had the option to repeat the module, adapt the difficulty, or continue with the next module. In total, 9 weekly modules were available to the participant. This was automatically registered. After some consideration, the research team had decided that completion of at least 6 modules was required to improve PA and other primary effects. Patients were defined as users if they completed at least 6 out of 9 modules. Participants who did not reach this threshold were defined as nonusers. Predictors of usage were collected through online baseline questionnaires and can be categorized as demographic, clinical, or psychological predictors. The potential predictors were not selected on theoretical grounds.

#### Demographic Predictors

Demographic predictors were gender, education (low: primary and lower vocational education; middle: secondary and middle vocational education; high: higher vocational and university education), and age (years) as demographic predictors.

#### Clinical Predictors

Clinical predictors in this study were location of OA (knee, hip or both), duration of OA complaints (years), and body mass index (BMI) (weight in kilograms divided by height in meters squared). Pain and fatigue were assessed on a 10-point Numerical Rating Scale (0 is no pain/not tired, 10 is worst possible pain/extremely tired). Self-reported PA was measured by the validated PA Scale for the Elderly (PASE) [31]. The PASE questionnaire is designed to assess PA patterns in older adults. The instrument consists of questions on household, leisure time, and work-related activities. Performance of the activities (assigned according to the level of intensity: light, moderate, and strenuous) is recorded as never, seldom (1-2 days/week), sometimes (3-4 days/week), or often (5-7 days/week). The amount of time spent in each activity is multiplied by its intensity. Physical functioning was determined by a subscale of the Knee OA Outcome Score (KOOS) [32,33] and the Hip Injury OA Outcome Score (HOOS) [34,35]. The KOOS and HOOS are self-administered questionnaires designed to assess patients’ opinions about their knee- and/or hip-related problems. The questionnaires assess 5 indicators on a 5-point Likert scale: pain, symptoms, physical functioning, sport/recreation functioning, and quality of life. The presence of self-reported comorbidity was obtained through a specific list of comorbid diseases. The list described the most prevalent chronic diseases and disorders in The Netherlands [36].

#### Psychological Predictors

Anxiety and depression were evaluated by a 14-item Hospital Anxiety and Depression scale [37]. Seven items on this questionnaire are related to anxiety and seven are related to depression. A lower score represents less anxiety and depression. Self-efficacy was evaluated by the Arthritis Self-Efficacy Scale for pain and other symptoms [38,39]. We used the subscales self-efficacy for pain and self-efficacy for other symptoms (eg, fatigue, depression). The score ranges from 1-10, where a higher score indicates greater self-efficacy.

Active and passive pain coping were determined by the Pain Coping Inventory questionnaire [40]. This 33-item questionnaire determines active and passive pain-coping strategies. A higher score on the active pain-coping subscale indicate a more adequate pain coping, and a higher score on the passive pain-coping subscale indicates inadequate pain coping. Locus of control, the extent to which one believes that one’s health is determined by one’s behavior, was examined with the Multidimensional Health Locus of Control Scale (MHLC) [41]. We used two subscales of the MHLC: (1) belief of control by powerful others (6 items) and (2) internal locus of control (6 items). For each subscale, a higher score indicates a greater level of belief in a particular subscale.

### Data Collection and Outcomes of the Qualitative Study

One year after being assigned to the program, a subgroup of participants from the intervention group was interviewed. All participants from the intervention group (n=100) were categorized into two groups: (1) users and (2) nonusers. Since the nonuser group showed considerable divergence in extent of program use (0 to 5 modules), we decided to invite more nonusers than users for our interview sample. This was executed by a stratified purposive sampling procedure [42]. After the stratified sampling, participants were contacted by phone, invited to participate, and scheduled for a face-to-face interview until the sampling goal was reached. The goal was to conduct 15 interviews (10 users and 5 nonusers). To reach this sampling goal, 24 participants were invited; 15 agreed to be interviewed and 9 decided not to participate due to a lack of interest. All participants who declined to be interviewed were nonusers. Semistructured interviews were conducted by the same interviewer (MB) in the respondents’ homes and lasted approximately 60 minutes. Interviews were digitally audiorecorded with the participants’ permission. The interviews were transcribed by means of the program Express Scribe [43]. During the interview process, we used an open-question guide (see Multimedia Appendix 2). This interview guide contained three topics: (1) patient characteristics, (2) intervention characteristics, and (3) study characteristics. The intervention characteristics contained three of the five themes described by Eysenbach’s law of attrition [17]: (1) *Relative advantage*, the degree to which the innovation is perceived to be superior to the ideas that it replaces [44], (2) *Complexity*, the degree to which an innovation is perceived as relatively difficult to understand and use [44], and (3) *Compatibility*, the degree to which an innovation is perceived as being consistent with the values, experiences, and needs of potential adopters [44].

### Analyses

#### Quantitative Analyses

Descriptive analyses were performed to describe participant characteristics and program usage. Logistic regression analysis with a stepwise backward selection procedure was used to build the most parsimonious prediction model. Program use (user/nonuser) was employed as a dichotomous dependent variable. Demographic, clinical, and psychological variables were the independent variables. Statistical analyses were conducted in two phases. First, potential predictors of interest were screened by univariate logistic regressions. Second, variables that achieved *P*<.20 were included in a multivariate stepwise regression analysis. Variables with the highest *P* value were removed one by one, until all remaining variables were *P*<.10. Only the final model was reported. Since this mixed methods study is explorative rather than hypothesis confirming, we decided to use the threshold value of *P*<.10. A sensitivity analysis was conducted to determine the robustness of usage thresholds. The sensitivity analysis was performed by changing the threshold of 6 modules to 5 modules (minus 1) and 7 modules (plus 1); this was subsequently repeated in univariate and multivariate analyses. Model fitting was evaluated with the Receiver Operating Characteristic (ROC) curve and the Hosmer-Lemeshow test. Statistical analyses were performed using SPSS Statistics 20.0.

#### Qualitative Analyses

Interviews were analyzed by means of deductive and inductive content analysis [42]. In the deductive approach, a template was created based on three concepts of Eysenbach’s law of attrition (relative advantage, complexity, and compatibility) [17]. Guided by these predetermined concepts, text sections were analyzed and coded. In addition to the deductive approach, an inductive method with no predetermined structure was employed. Based on the grounded theory approach [45], recurrent themes from the interview data were identified, coded, labeled, and grouped into broader concepts. While the deductive “top-down” approach tests pre-existing concepts of (non)usage, the inductive “bottom-up” approach starts with patterns observed from the interview data. Data analysis was performed using the software MAXQDA [46] for textual analysis. All interviews were analyzed by the researcher (MB). To assess interrater reliability, a random sample of five interviews was analyzed by a second investigator (DB). Codes were compared and disagreements were resolved by discussion between the 2 researchers. No major differences were found in codes between the 2 researchers.

## Results

### Quantitative Results

#### Program Completion

**Figure 2:**
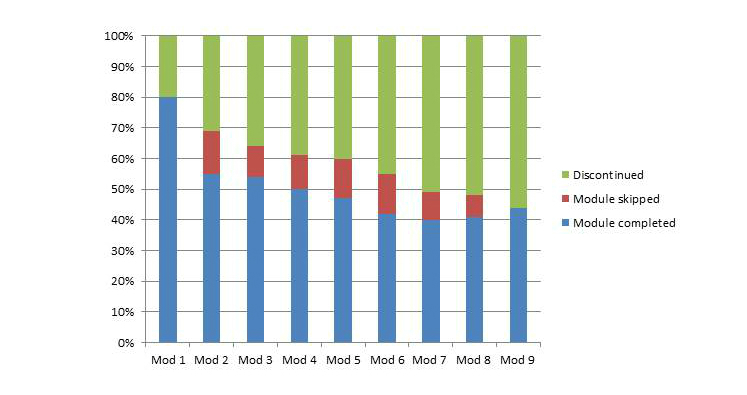
Program use.

Of the 100 participants who received a password and username to enroll, 49 users made a start with the first module and 6 participants never logged in to their personal website. Figure 2 depicts an overview of the module completion rate; 80% of the subjects completed the first module. This percentage declined to 55% during the second module. Approximately 50 of the 100 users completed modules 3, 4, 5, 6, 7, 8, and 9. The average number of modules completed was 5.6 (SD 2.9) out of 9 modules. Since personal messages were updated on a weekly basis, patients had the opportunity to complete a module within 7 days. Overall, 19 of the 100 participants completed all modules of the program, and 46 of the 100 users used at least 6 out of 9 modules. Consequently, 46 participants were defined as users and 54 as nonusers. Users finished a median of 8 (SD 1.1) modules and nonusers a median of 2 (SD 1.5) modules. Adverse events, such as extreme pain and injuries, were not reported during the program.

#### Predictors of Program Usage

presents demographic, clinical, and psychological baseline variables for users and nonusers. Univariate analyses showed that age, BMI, symptoms, and comorbidity reached the threshold of *P*<.20. Based on these variables, three multivariate models were built, which resulted in the most parsimonious predictors including age and comorbidity (Table 2). Higher age (*P*=.08, OR 0.94) and presence of comorbidity (*P*=.02, OR 0.33) were negative predictors for program completion. The sensitivity analysis indicated that the model was robust to changes in the parameter usage. The area under the ROC curve for the model was .68 (95% CI 0.57-0.79). The Hosmer-Lemeshow test of goodness of fit was not statistically significant (*P*=.43), indicating that the data fitted the model well.

### Qualitative Results

#### Overview

The qualitative deductive and inductive analysis resulted in the identification of several reasons for (non)usage. The majority of reasons were found by the deductive analysis. Additionally, the inductive analysis identified a number of personal factors (eg, social environment and emotional factors) relating to (non)usage. Reasons are divided into patient, intervention, and study characteristics and are illustrated by interview quotes. Additional quotes illustrative of each theme are provided in Multimedia Appendix 3.

#### Patient Characteristics

Interviewees reported that a low mood interfered with their ability to perform modules. One participant summarized this sentiment by saying, “I had a bad year and I was not at ease with myself. I was not in the right mood to exercise. It was all too much” [woman, hip OA, nonuser]. Lack of self-discipline was another identified reason for nonusage. As one man put it “This kind of program does not work for me. I find it difficult to stay motivated all the time. At the beginning I was motivated but then it went downhill quickly. I got lazy and other activities became more important” [man, knee OA, nonuser]*.* Another reason for discontinuation was the presence of an additional health problems, other than OA. Due to pain and/or other (medical) treatments, it was difficult for interviewees to continue their involvement in the *Join2move* program. In addition, participants who regarded themselves as already physically active found it less necessary to participate. By contrast, patients who felt themselves responsible for their own progress were most likely to use the program. These individuals perceived the program as something that needed to be done, rather than appreciation or enjoyment. Furthermore, those who emphasized the importance of their partner, family, or friends in maintaining the *Join2move* program were mostly adherent. One participant commented: “Regularly, my husband and friends joined me because I told them about the program. This motivated me to continue” [woman, knee OA, user]*.*

#### Intervention Characteristics

Participants reported that several characteristics of the *Join2move* intervention were identified as a reason for (dis)continuation. Overall, they expressed positive feedback regarding the complexity of the program*.* Usability problems with respect to the functionality of the website were not reported. The values “trust” and “reliability” were important in the decision to engage the *Join2move* program. To cite one patient: “Join2move is based on an evidence-based theory. This persuaded me to participate and to continue with the program” [man, knee OA, user]. Further, patients consistently reported that the Web-based character of the intervention was an advantage compared with face-to-face treatments. The flexibility of being able to complete modules at one’s own pace without time or travel restrictions was cited as an advantage. On the other hand, the Web-based character also had a downside. Some participants had a strong need for personal guidance. In the words of one participant: “Although it was possible to fill out an evaluation form about pain and performance, sometimes I just needed a personal chat to talk about my progress” [man, knee OA, nonuser]. Moreover, gradually increasing a self-selected activity was not always compatible with expectations. As one participant said: “I expected a package of specific exercises instead” [woman, knee OA, nonuser].

#### Study Characteristics

Study-related factors were also cited as reasons for remaining or not remaining engaged in the program. Some participants felt under obligation to continue. They described a feeling of commitment to the organizers of the study. “Because I was allocated to the intervention group, I wanted to finish the entire program. Maybe a little old-fashioned but I found it inappropriate to stop halfway” [woman, knee OA, user]. Some participants perceived the questionnaires used as being too long or too difficult. The questionnaire consisted of 17 pages with a total of 171 items. Participants not only lost interest in completing the questionnaires but were also less motivated to continue with the program.

**Table 1:** Baseline demographic and clinical characteristics.

			Users, n=46	Nonusers, n=54	*P* value
**Demographic predictors**					
	**Gender, n (%)**				
		Male	17 (37)	23 (43)	.57
		Female	29 (63)	31 (57)	
	Age (years), mean (SD**)**		60 (6.3)	62 (6.5)	.09
	**Education**				
		Lower education	7 (15)	6 (11)	.60
		Middle education	18 (39)	18 (33)	.41
		Higher education	21 (46)	30 (56)	.42
**Clinical predictors**					
	**Location OA, n (%)**				
		Knee	30 (65)	36 (67)	.89
		Hip	11 (24)	11 (20)	.80
		Both	5 (11)	7 (13)	.64
	OA duration (years), mean (SD)		2.8 (1.3)	2.8 (1.1)	.86
	**BMI (kg/m** ^**2**^ **), n (%)**				
		Normal weight (<25)	22 (48)	17 (31)	.10
		Overweight (>25)	24 (52)	37 (69)	
	**Comorbidity, mean (SD)**				
		No, n (%)	36 (78)	30 (56)	.02
		Yes, n (%)	10 (22)	24 (44)	
	Physical activity		117 (66.1)	130 (65.5)	.29
	Pain, 0-10		5.4 (2)	5.4 (2.3)	.92
	Fatigue, 0-10		4.7 (2.7)	5.2 (2.8)	.34
	Symptoms		56 (15.6)	60 (17.8)	.17
	ADL		58.3 (22.3)	55.3 (19.9)	.47
	Sport and recreation		58 (22)	55 (19.9)	.47
	Quality of life		38.7 (16.9)	42 (17.4)	.32
**Psychological predictors, mean (SD)**					
	Self-efficacy pain		3.4 (0.8)	3.4 (0.9)	.67
	Self-efficacy other symptoms		3.5 (0.9)	3.4 (0.9)	.60
	Active pain coping		2.0 (0.4)	2.1 (0.4)	.34
	Passive pain coping		1.8 (0.4)	1.9 (0.4)	.26
	Anxiety		4.7 (3)	4.5 (2.9)	.62
	Depression		3.8 (2.9)	3.8 (3)	.88
	Internal locus of control		23 (5.4)	23.7 (4.3)	.46
	Powerful others locus of control		15.3 (4.4)	15.9 (4.5)	.54

**Table 2:** Univariate and multivariate analyses for predictors for usage^a^.

		B^b^	SE^c^	OR (95% CI)	*P* value
**Univariate analyses**					
	Age, years	−.06	.04	.94 (0.88-1.01)	.09
	BMI (normal weight/overweight)	−.69	.42	.50 (0.22-1.13)	.10
	Comorbidity (no/yes)	−.93	.44	.39 (0.14-0.84)	.02
	Symptoms (0-100)	−.02	.01	.98 (0.96-1.01)	.17
**Multivariate analyses**					
	Age, years	−.07	.04	.94 (0.87-1)	.08
	Comorbidity (no/yes)	−1.1	.46	.33 (0.13-0.82)	.02

^a^The reference groups are nonusage, normal weight, and no comorbidity.

^b^B=beta coefficient.

^c^SE=standard error.

## Discussion

### Principal Findings

The aim of this mixed methods study was to identify patient, intervention, and study characteristics that facilitate or impede the usage of a Web-based intervention for patients with knee and/or hip OA. Results from this study showed that participants with knee and/or hip OA used the *Join2move* program less than intended. Of all participants, 94% started the program, 46% reached the threshold of 6 out of 9 completed modules, and 19% finished all 9 weekly modules. To put these rates into perspective, we refer to Hansen et al [7] who found that merely 7% of inactive participants logged in once to a self-guided Web-based PA intervention, and Irvine et al [8] showed that 46% of the users completed all 12 sessions of a self-guided Web-based PA intervention. In a study among patients with rheumatoid arthritis, Van den Berg et al [47] reported that 86% of the patients assessed a website once per week for the duration of 3 months. When considered in light of these studies, our usage rates can be interpreted as reasonable. However, Web-based interventions differ widely in terms of population, content, setting, and methods of measuring usage. For example, while our study used number of modules completed for measuring usage, the above-mentioned studies used log-in data [7,8] or questionnaires [47] as measures. Further, our intervention was self-directed, while the program by Van den Berg et al [47] contained supervision. These differences may have had a major impact on usage and indicates that direct comparison with other reported Web-based interventions remains difficult. In an effort to overcome this issue, the systematic review by Kelders et al [26] adopted the concept of intended usage. This is a universal measure for adherence, which is defined as the extent to which users should experience the content of the intervention to derive maximum benefit.

Considering the predictors of usage, it appeared from the quantitative analysis that age and comorbidity proved to be significantly related to program usage. Younger participants were more likely to use the intervention modules than older participants. This is in contrast to previous studies that have found correlations between older age and higher usage rates [9,21,22]. This discrepancy in findings can be explained by the fact that the mean age of our study sample was significantly higher (62 years) than the mean age of the other studies (42, 44, and 39 years respectively) [9,21,22]. In fact, the younger participants from our sample should be compared with the older subjects from other studies. This suggests that participants aged roughly 50-60 years are most adherent to Web-based interventions. Apart from this, the presence of an additional medical condition increased the odds of not using *Join2move*. These results were also confirmed in the interviews. Patients mentioned that physical discomfort during PA and specific comorbid-related factors such as pain, medication use, and disease-related constraints hampered their program performance. Another explanation might be that the program was solely focused on OA and no attention was paid to additional diseases. Participants with an additional illness might feel that the *Join2move* program did not suit their needs. Unfortunately, it was not possible to examine the influence of each comorbidity on usage due to the low number of cases per disease category. Further research is required to examine which of the comorbidities is most predictive in relation to (non)usage.

With respect to the intervention, participants indicated that the automatic gradual increase of PA as well as working toward a short-term goal were mechanisms that supported them in completing weekly modules. Compared with face-to-face treatments, the flexibility of completing modules at one’s own pace without time or travel restrictions was cited as a major advantage. However, older patients, those with comorbidity and patients who attach great importance to personal contact indicated that the lack of human involvement was a disadvantage. Furthermore, from the interviews it became clear that those who felt themselves responsible for their own progress were most likely to use the program. This, however, was not confirmed in the quantitative analysis. Although we included questions about responsibility and persistence, the questionnaires were not sensitive enough to confirm the conclusions from the qualitative analysis. This illustrates very well why we have chosen dual data collection. The weakness of questionnaires was compensated by interview data. Other mentioned motivations for (non)usage were trial specific. While questionnaires impede usage, commitment to the research team was described as an important facilitator for usage. We did not find any predictive value for education and gender, in contrast to other studies [22,24].

### Limitations

A major weakness is the potential presence of recall bias. In an effort to prevent attention bias during the previously conducted randomized controlled trial, the length of time between program participation and interviews was approximately 12 months. As a consequence, participants may not have accurately remembered the intervention in detail. This may have affected the reliability of our results. Another weakness is that results are limited in their generalizability because participants were mainly older, healthy, and highly educated patients with knee and/or hip OA. Furthermore, the role of motivation as proximate determinant of usage behavior was not investigated in this study. Future research should examine the role of motivation on program usage. A last limitation was that participants were included on the basis of self-reported OA. Diagnosis was not confirmed through clinical tests or x-ray reports due to practical reasons. Although self-reported OA is a common inclusion strategy in the field of osteoarthritis research, it is presumable that we have included false positive OA patients in the study.

### Future Directions and Implications

In light of rising health care costs and the large population of patients with knee and/or hip OA, *Join2move* is an effective, low-cost, and promising program for improving PA levels in patients with knee and/or hip OA. We believe that the quantitative and qualitative results provide insights that are of relevance to the field of Web-based health education. Future Web-based PA programs should include gradual activity programs with attainable short-term goals. Goal setting, preferably by participants themselves, as well as feedback on performance seem to be powerful tools for increasing the usage of Web-based interventions. Future studies should also pay special attention to older patient groups and patients with a comorbid condition. For these groups a more personal approach is needed. In a further study, we will investigate if guidance by a physical therapist will lead to higher levels of usage. The fact that participants described a feeling of commitment to the organizers of the study may indicate that observed usage patterns cannot be replicated in a real-life setting. Conducting more practically oriented research is an important way to explore usage rates in real-world settings.

## Multimedia Appendix 1

Screenshots intervention.

## Multimedia Appendix 2

Interview guide.

## Multimedia Appendix 3

Interview quotes.

## Abbreviations

BGA: behavior graded activity
BMI: body mass index
HOOS: Hip Injury OA Outcome Score
KOOS: Knee OA Outcome Score
MHLC: Multidimensional Health Locus of Control Scale
OA: osteoarthritis
OR: odds ratio
PA: physical activity
PASE: PA Scale for the Elderly
RCT: randomized controlled trial
ROC: Receiver Operating Characteristic
