# Supplementary material for: Adherence to a Web-Based Physical Activity Intervention for Patients With Knee and/or Hip Osteoarthritis: A Mixed Method Study
Source: J Med Internet Res. 2013 Oct 16;15(10):e223. doi: 10.2196/jmir.2742 (PMC3806355; doi:10.2196/jmir.2742)

Homepage

Artrose in Beweging.nl

Persoonlijk beweegprogramma

nivel

E-mailadres ...

••••••••••••••

Inloggen

☐ Laat mij aangemeld blijven

Home | Over artrose | Leven met artrose | Programma & Onderzoek | Contact

Inschrijven voor het programma?

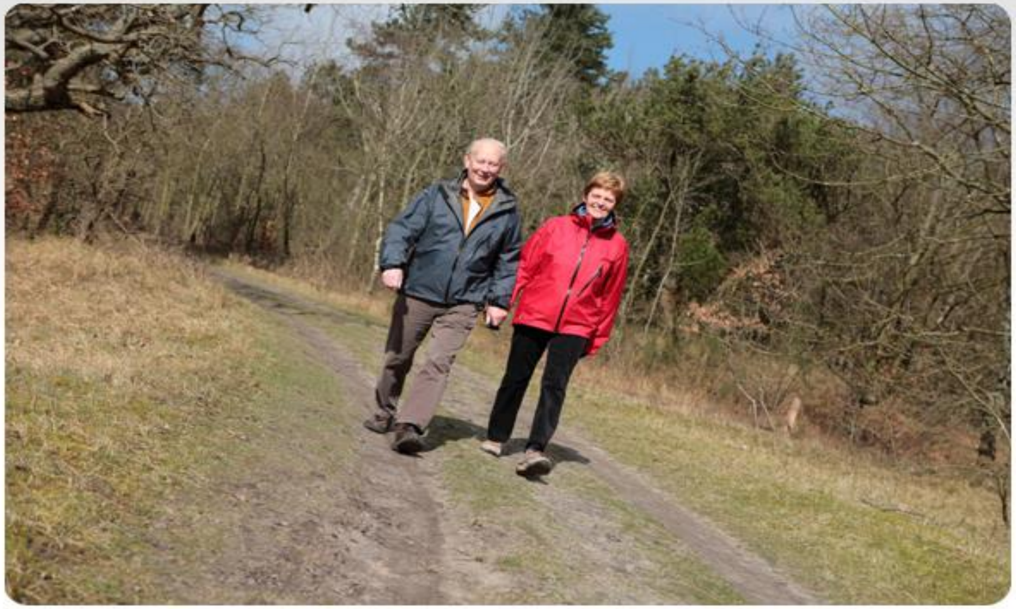

Meneer Verveer (61) - *"Artrose In Beweging was voor mij de stok achter de deur om meer te bewegen. Ik vind het heerlijk om samen met mijn vrouw te wandelen en te genieten van de natuur"*

Bewegen helpt!

Gedoseerd bewegen houdt bij mensen met artrose de conditie op peil, spieren sterk, gewrichten soepel en geest gezond. Hierdoor worden dagelijkse activiteiten, zoals wandelen, tuinieren en fietsen gemakkelijker uitvoerbaar.

Bent u tussen de 50 en 70 jaar, heeft u last van artrose in de heup en/of knie en wilt u stapsgewijs meer gaan bewegen? Schrijf u dan in voor het beweegprogramma.

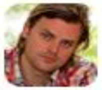

Met vriendelijke groet,
Daniël Bossen

Start onderzoek februari 2011
In februari start het NIVEL met een onderzoek naar de effectiviteit van het internet beweegprogramma. De inschrijving voor het onderzoek is verlengd tot en met juni 2011! Schrijf u hier in!

Artrose in Beweging.nl © 2010

Disclaimer - Inschrijven voor het beweegprogramma? - Contact

Stage 2 of introduction: Selection of central physical activity

Artrose in Beweging.nl

Persoonlijk beweegprogramma

nivel

Aangemeld als datfa

Mijn Profiel | Uitloggen

Mijn beweegprogramma | Thuisoefeningen | Leven met artrose | Over artrose | Contact | Werkboek

- Introductie
- 1. Gezondheidsvragen
- **2. Centrale activiteit**
- 3. Basismeting
- 4. Korte- en lange termijndoel
- 5. Bewegen met pijn
- 6. Beweegovereenkomst

Kiezen van activiteit

Dit programma duurt 8 weken waarbij een activiteit centraal staat. Kies één activiteit, dus niet meerdere, die u stapsgewijs wilt opbouwen. De gekozen activiteit moet voldoen aan de volgende drie voorwaarden:

- De activiteit **moet leuk zijn** om te doen!
- U heeft **moeite met het uitvoeren** van de activiteit vanwege pijn, stijfheid of een verminderde conditie.
- De activiteit kan **drie keer** in de week uitgevoerd worden.

- ( ) wandelen, lopen
- ( ) zwemmen
- (•) fietsen
- ( ) gymnastieken
- ( ) dansen
- ( ) huishoudelijke taken (schoonmaken, stofzuigen, boodschappen doen)
- ( ) tuinieren
- ( ) roeien
- ( ) golfen
- ( ) schaatsen
- ( ) tennissen
- ( ) nordicwalking

Opslaan

Stage 3 of introduction: A 3-day self-test

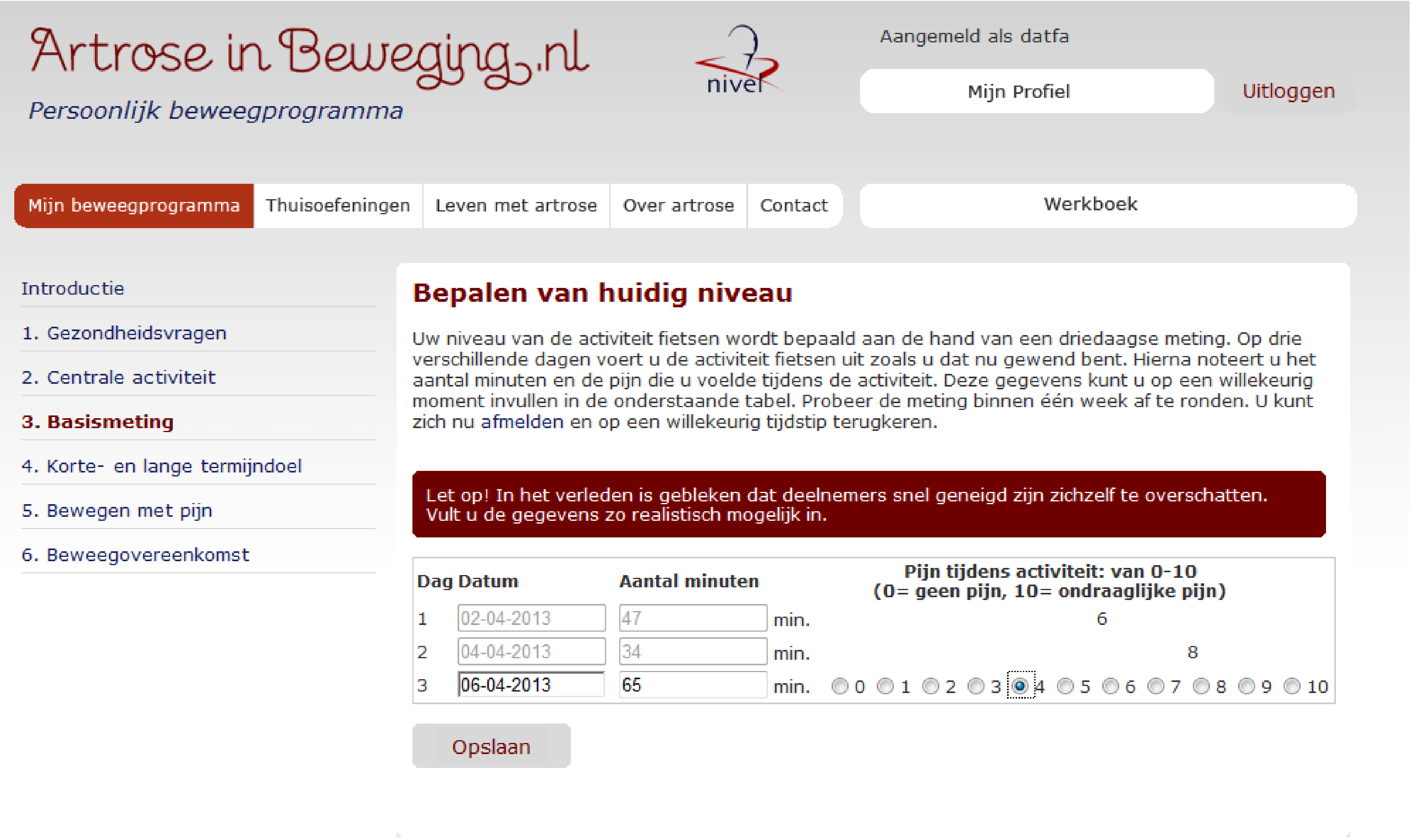

Stage 4 of introduction: Determining a short term goal

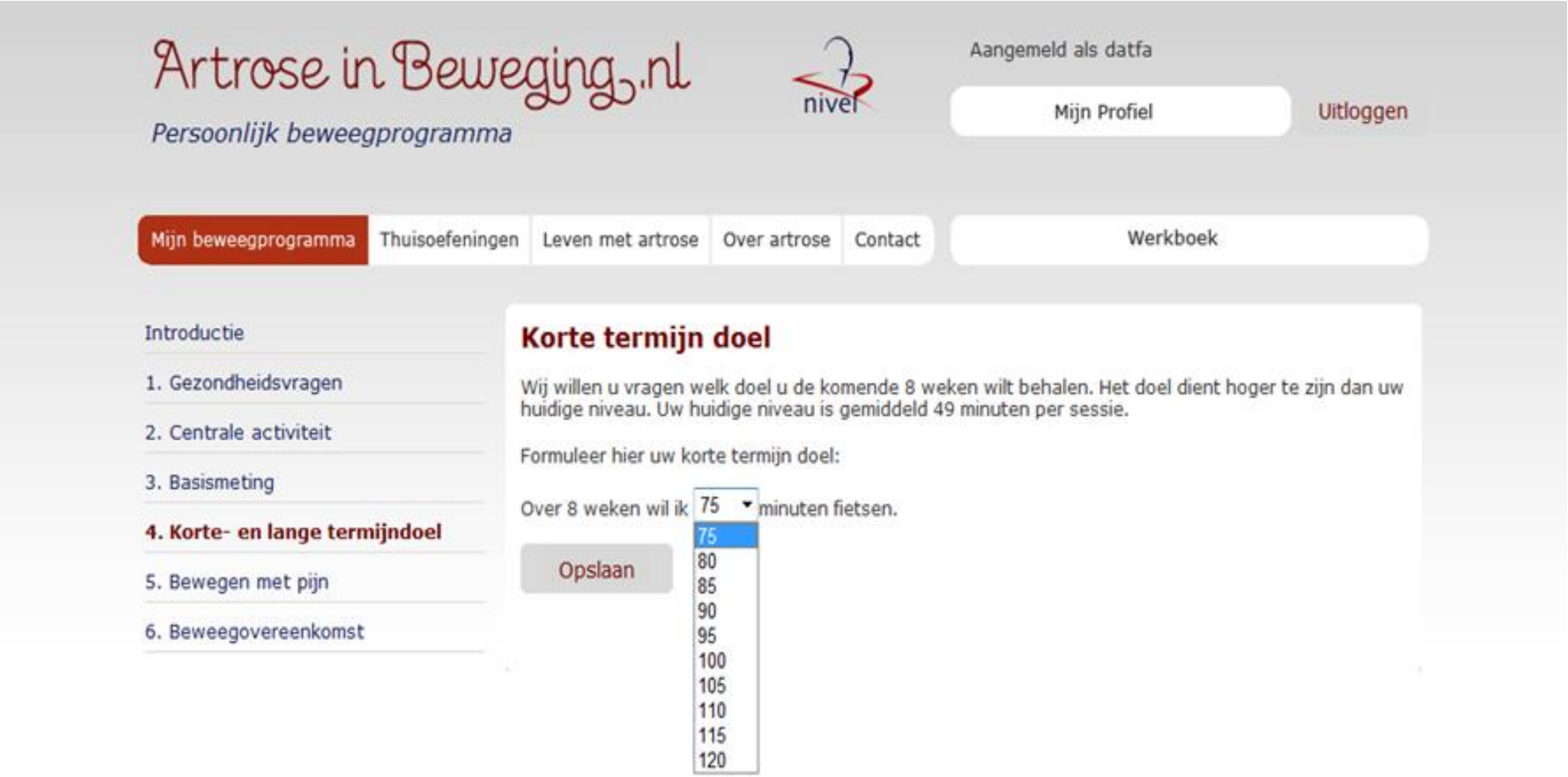

Stage 6 of introduction: Summary and agreement form

Artrose in Beweging.nl

Persoonlijk beweegprogramma

nivel

Aangemeld als datfa

Mijn Profiel | Uitloggen

Mijn beweegprogramma | Thuisoefeningen | Leven met artrose | Over artrose | Contact | Werkboek

- Introductie
- 1. Gezondheidsvragen
- 2. Centrale activiteit
- 3. Basismeting
- 4. Korte- en lange termijndoel
- 5. Bewegen met pijn
- **6. Beweegovereenkomst**

Beweegovereenkomst

Wij hebben uw doel in een beweegovereenkomst gezet. Uw doel voor dit programma is als volgt geformuleerd:

"**Over acht weken wil ik 75 minuten kunnen besteden aan fietsen**"

Om dit doel te bereiken krijgt u wekelijks een activiteitenopdracht. Tijdens deze opdrachten wordt uw niveau van de activiteit fietsen stapsgewijs opgebouwd.

Verder willen wij het volgende met u afspreken:

- U bent op de hoogte van uw persoonlijke korte termijn doel zoals hierboven is geformuleerd.
- Als u meer gaat bewegen kunt u meer pijn krijgen in spieren en gewrichten. Dit zijn gezonde reacties van het lichaam. Deze pijn is vervelend, maar **niet** gevaarlijk.
- U houdt zich, ondanks de pijn, aan de opdracht.

☑ Ik ga akkoord met bovenstaande beweegovereenkomst.

Opslaan

The first of eight week modules

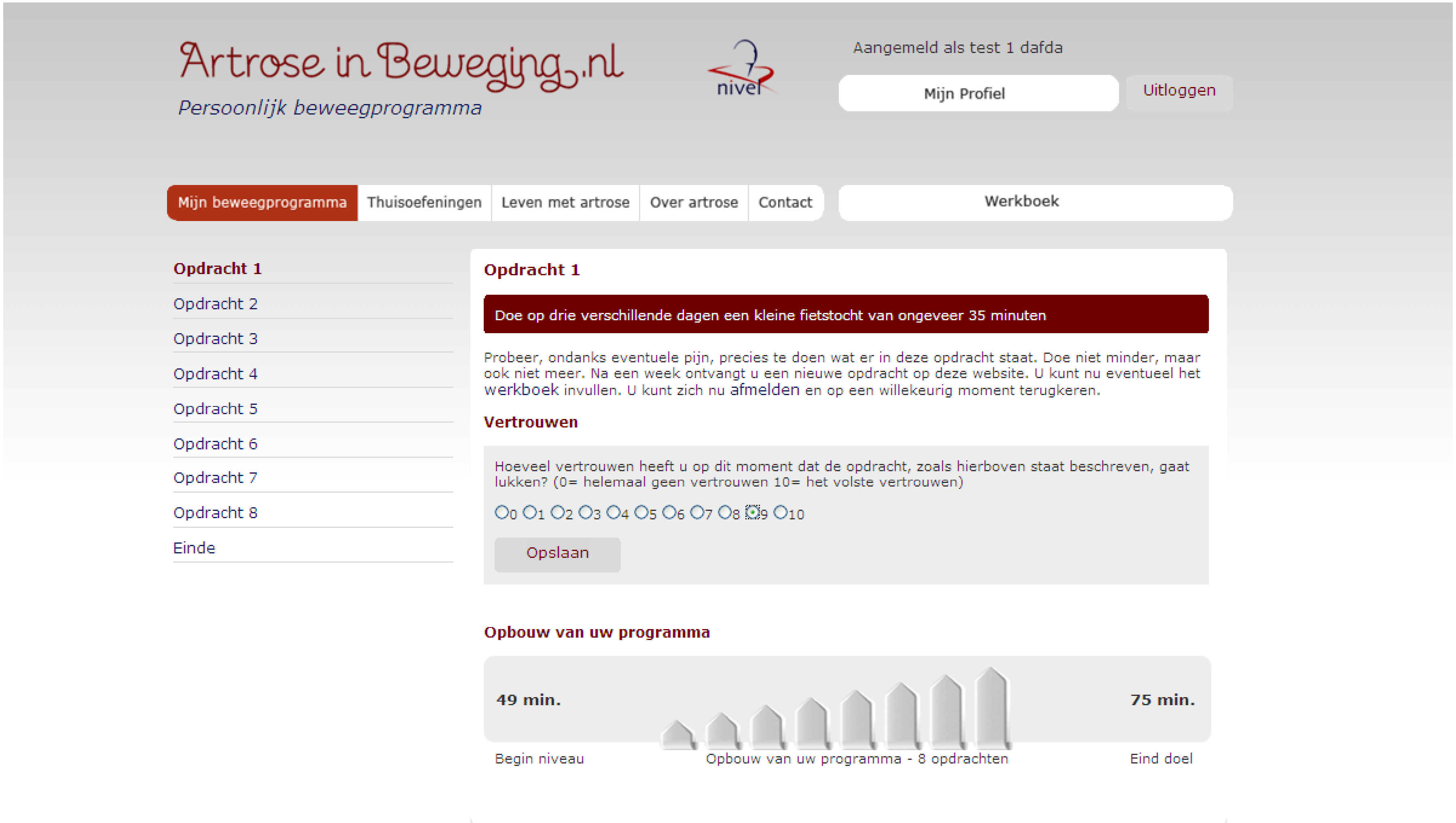

Evaluation form module 1

Artrose in Beweging.nl

Persoonlijk beweegprogramma

nivel

Aangemeld als datfa datfa

Mijn Profiel | Uitloggen

Mijn beweegprogramma | Thuisoefeningen | Leven met artrose | Over artrose | Contact | Werkboek

- Opdracht 1
- **Opdracht 2**
- Opdracht 3
- Opdracht 4
- Opdracht 5
- Opdracht 6
- Opdracht 7
- Opdracht 8
- Einde

Evaluatie van opdracht 1

Voordat u start met de volgende opdracht vragen wij u de vorige opdracht te evalueren. Uw opdracht afgelopen week was "Doe op drie verschillende dagen een kleine fietstocht van ongeveer 35 minuten"

Kunt u aangeven in hoeverre u deze opdracht heeft uitgevoerd?

- (●) Ik heb **precies** gedaan wat in deze opdracht staat aangegeven.
- ( ) Ik heb **minder** gedaan dan deze opdracht.
- ( ) Ik heb **meer** gedaan dan deze opdracht.

Vrije tekst (optioneel): Hoe is het gegaan met uw eerste opdracht?

Pijn

Hoeveel pijn heeft u gemiddeld ervaren tijdens opdracht 1?
(0= geen pijn, 10= ondraaglijke pijn)

( ) 0 ( ) 1 ( ) 2 ( ) 3 ( ) 4 (●) 5 ( ) 6 ( ) 7 ( ) 8 ( ) 9 ( ) 10

Opslaan

Opbouw van uw programma

49 min.

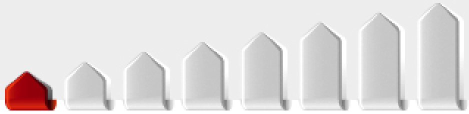

75 min.

Begin niveau — Opbouw van uw programma - 8 opdrachten — Eind doel

Information about OA and lifestyle

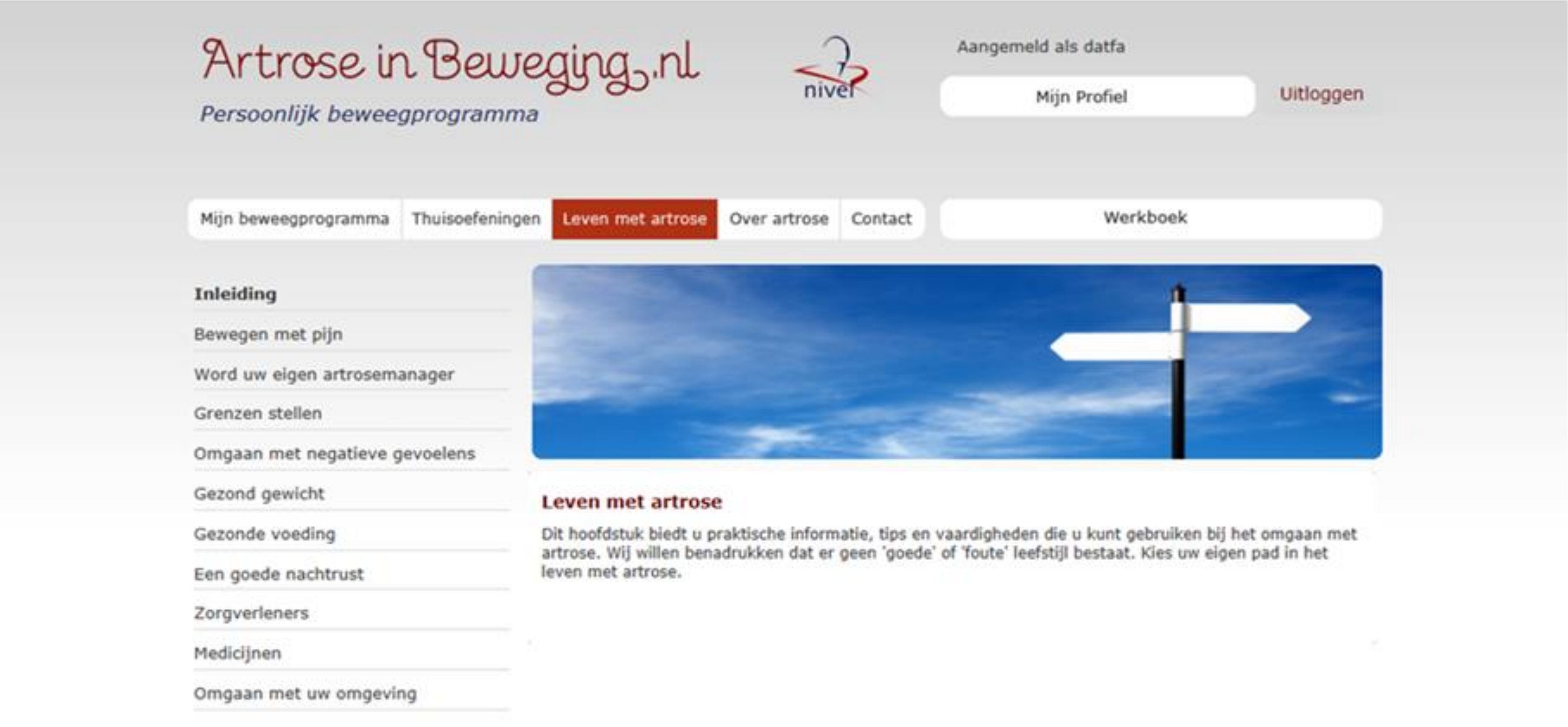

Additional strength and mobility exercises

Artrose in Beweging.nl
Persoonlijk beweegprogramma

nivel

Aangemeld als datfa

Mijn Profiel | Uitloggen

Mijn beweegprogramma | Thuisoefeningen | Leven met artrose | Over artrose | Contact | Werkboek

- Inleiding
- Spierkracht knie - oefening 1
- Spierkracht knie - oefening 2
- Spierkracht knie - oefening 3
- Spierkracht heup - oefening 4
- **Bewegelijkheid knie en heup - oefening 5**
- Spierkracht heup - oefening 6
- Spierkracht knie - oefening 7
- Spierkracht heup - oefening 8

Oefening 5: Bewegelijkheid knie en heup

Uitgangshouding:
Ga op uw rug liggen. Buig het been waar u niet mee oefent en strekt het te trainen been.

Uitvoering:
Buig uw gestrekte knie en schuif uw hiel naar uw billen toe. Zorg ervoor dat uw voeten contact blijven houden met de vloer. Wanneer u de voet zo ver mogelijk naar uw billen heeft gebracht trekt u met beide handen uw knie naar uw borst. Houd deze houding een vijftal seconden vast en breng vervolgens uw knieweer in strekpositie. Herhaal deze oefening 8-12 keer, neem even rust en doe dit dan nog twee keer. Dus drie series van 8-12 herhalingen. Herhaal dit ook met uw andere been.

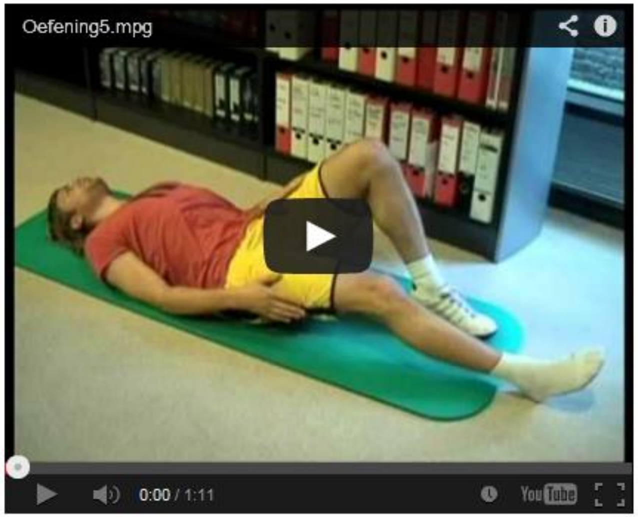

Supplement: Supplementary file 1 [file jmir_v15i10e223_app1.pdf]
