# Supplementary material for: Adherence to a Web-Based Physical Activity Intervention for Patients With Knee and/or Hip Osteoarthritis: A Mixed Method Study
Source: J Med Internet Res. 2013 Oct 16;15(10):e223. doi: 10.2196/jmir.2742 (PMC3806355; doi:10.2196/jmir.2742)
Supplement: Supplementary file 2 [file jmir_v15i10e223_app2.pdf]

Interview guide

Themes	Examples general questions	Examples of prompts
Introduction	Aim of interview<br>Permission recording<br>Information privacy policy	
Participant characteristics	Social environment<br>Problems related to OA/PA<br>Experiences<br>Expectations	- What did you expect when you decided to participate *Join2move*?<br>- Why you have chosen to participate in this program?<br>- Were other people aware/involved during your participation?
Intervention characteristics	General aspects<br>Reasons (non)usage<br>Relative advantages<br>Complexity<br>Compatibility	- Overall, what did you like most about Join2move? Influence on usage?<br>- What did you like the least? Influence on usage?<br>- Did you experience difficulties? (language, functionality etc.) Influence usage?<br>- Do you have any suggestions for improving the Join2move program?<br>- Did you participate in an (online) PA program before?<br>- It was not possible to test out the program, would practicing improve the program? Influence usage??
Study characteristics	Information letter/informed consent<br>Questionnaires<br>Impact study participation	- Did you experience any difficulty in completing the online questionnaires? Influence usage?<br>- Why have you decided to participate in a the study?
Ending	Summary interview	

OA; Osteoarthritis, PA; Physical Activity.
