# Supplementary material for: Adherence to a Web-Based Physical Activity Intervention for Patients With Knee and/or Hip Osteoarthritis: A Mixed Method Study
Source: J Med Internet Res. 2013 Oct 16;15(10):e223. doi: 10.2196/jmir.2742 (PMC3806355; doi:10.2196/jmir.2742)
Supplement: Supplementary file 3 [file jmir_v15i10e223_app3.pdf]

Interview quotes

Patient characteristics

Comorbidity

"A while after registration I began to suffer from tendinitis in my right foot. During the first module the foot was so painful that I decided to quit" [woman, knee and hip OA, non-completer].*" In the year that I registered, I was diagnosed with prostate cancer. I have had surgery and received radiotherapy treatments for several months. Therefore using the Join2move program was too hard for me."* [man, knee OA, non-completer].

Well-being

" I had a bad year and I was not at ease with myself. I was not in the right mood to exercise. It was all too much" [woman, hip OA, non-completer]

Social support

"*My husband and friends joined me regularly because I told them about the program. This motivated me to continue.* [woman, knee OA, completer]

Already physically active

"I have a fulltime job and walk around the office all day. So for me it was not necessary to walk the extra miles for the Join2move program." [man, knee OA, non-completer]

Lack of motivation

"*This kind of program does not work for me. I find it difficult to stay motivated all the time. At the beginning I was motivated but then it went downhill quickly. I got lazy and other activities became more important*. [man, knee OA, non-completer]

Sense of duty

"Although it was a virtual person, I made an agreement and if I make an agreement I stick to it."[man, hip OA, completer]

Intervention characteristics

Trust and reliability

"Join2move is based on an evidence based theory. This persuaded me to participate and continue with the program" [man, knee OA, user]." *The content and feedback of the system was put together well. This made me feel confident that I was in good hands."* [man, knee OA, user].

Usability and complexity

"The language used in the program was easy to understand and appealing" [man, knee OA, non-user].*"Although it was quite simplistic, the structure of the program was an effective and appropriate way to increase my physical activity level"* [man, knee OA, user].

Advantages and disadvantages

"The internet aspect of the program was very convenient. It was not necessary to go out for a weekly appointment. That saved me a lot of time. [woman, hip OA, completer.]

"*Although it was possible to fill out an evaluation form about pain and performance, sometimes I just needed a personal chat to talk about my progress"* [man, knee OA, non-completer].

Expectations about the program

"I expected a package of specific exercises instead of performing 'all day' activities"[woman, knee OA, non-completer].

Study characteristics

Commitment to the researchers

”Because I was allocated to the intervention group, I wanted to finish the entire program. Maybe a little old fashioned but I found it inappropriate to stop halfway. [woman, knee OA, completer]

Questionnaires

“*The questionnaires included too many questions and some questions were hard to answer. Eventually I didn’t want to make the effort anymore, so I decided to quit the program.”*[woman, knee OA, non-completer].
